# The Role of Trait Empathy and State‐Level Emotion in Predicting Helpers' Facilitative Interpersonal Skill

**DOI:** 10.1002/jclp.70112

**Published:** 2026-02-19

**Authors:** Timothy Anderson, Tao Lin, Hillary Benanzer, Suzannah J. Stone, Kim de Jong

**Affiliations:** ^1^ Ohio University Athens Ohio USA; ^2^ Institute of Psychology Leiden University Leiden Netherlands

**Keywords:** emotional expression, facilitative interpersonal skills, responsiveness, therapist characteristics

## Abstract

**Objective:**

Common factor, interpersonal, and dynamic therapies assume that therapists' trait empathy and in‐the‐moment emotional experiences play a central role in therapeutic processes. This study tested whether a trait level predictor (empathy) and three state‐level emotion predictors (anxiety, positive affect, negative affect) could predict Facilitative Interpersonal Skills (FIS).

**Method:**

A total of 96 participants who self‐identified as having an interest in becoming therapists responded to provocative, interpersonally difficult client simulations from the Facilitative Interpersonal Skills task (FIS) as well as a companion set of simulations that were selected to be benign (i.e., less challenging). Helpers completed measures of state‐level emotions immediately after the clips. Ratings of FIS were made by both the participants and independent observers.

**Results:**

FIS ratings (Skill) were highly correlated on both difficult and benign clips. Therapist positive affect (but not negative affect) was predictive of observer‐rated FIS, whereas anxiety was predictive of helper‐rated FIS.

**Discussion:**

Helpers may associate the ability to modulate anxiety with their self‐judgments of being skillful in challenging therapeutic situations. However, trained observations of these skills were associated with helper experiences of positive affect (and not anxiety or negative affect). One implication of this finding is that negative and positive affect may play different roles for how skill is perceived within students learning therapeutic skills versus observers (e.g., supervisors).

Throughout the history of psychotherapy, considerable research and theory has been built around the notion that therapists' emotional and interpersonal reactions to clients, particularly negative emotional reactions, have salient effects on treatment (e.g., Strupp and Binder 1984; Safran and Muran 2000; Eubanks‐Carter et al. 2015). Though overt hostility by therapists is relatively rare, the documented impacts of such countertransference (Hayes et al. 2018) on both in‐session interpersonal processes—for example, in leading therapists to engage in more interpretations about trait‐like characteristics of their clients and fewer reflections of their feelings (Anderson et al. 2012)—and overall clinical outcomes (Henry et al. 1990) warrant further attention.

Even with these high stakes, there has been relatively little research that unpacks difficult interpersonal moments, and there is practically no research about what happens *after* clients say something that could potentially stimulate a negative emotional reaction from their therapists. Obstacles to obtaining knowledge about therapists' negative reactions stem from the responsiveness problem in psychotherapy (Stiles 2021) and the slow development of technology that could allow for observation in these moments. First, the responsiveness problem refers (partly) to the fact that communication is complicated, and real interpersonal communication takes place within an emerging context, co‐created by a complex history of prior messages. This means that no two relationships are the same, thus no two therapist‐client dyads are comparable (Stiles 2021). The problem posed by unique emergent contexts in relationships is relevant to the second problem of finding methodological solutions to control or standardize these infinitely variable difficult therapy moments, such that all therapists' emotional reactions are not confounded by other variables within the context.

The Facilitative Interpersonal Skills research task (FIS; Anderson and Perlman 2022) was designed to address some of these methodological problems. The task experimentally manipulates a variety of brief, difficult simulated situations with clients as a way to stimulate the therapist/helper during moments when they are providing an intervention to the simulated client. These video simulations are brief (approximately 1 min each in length) and consist of difficult interpersonal scenarios drawn from real, prior therapy sessions. Participants' responses to the stimulus clips are video recorded and then rated by trained observers for their display of eight facilitative skills: fluency, emotional expression, persuasiveness, warmth/positive regard, hopefulness, empathy, alliance bond capacity, and alliance‐rupture‐repair responsiveness (Anderson et al. 2009; Anderson et al. 2020). The standard, performance‐based procedure of the FIS task minimizes self‐report bias and systematically controls client‐related variability.

Therapists who express higher amounts of common interpersonal skills (e.g., empathy, expressed warmth) toward these standardized, difficult clients have been shown to have higher symptom improvement with their clients (Anderson et al. 2016; Anderson et al. 2016; Anderson et al. 2009). While there are encouraging findings on therapist FIS and client outcomes, the subject of *how* the emotional experiences of therapists may be associated with FIS remains largely unstudied. Anderson et al. (2020) postulated that therapists' internal processing of negative, interpersonal emotions to the clients in the FIS stimulus clips may play a central role in understanding how interpersonal skill are generated. Therapists who are adept at managing negative emotional reactions during difficult client communications are presumed to be more skillful, and the FIS task presents therapists with stimuli that are designed to stimulate negative feelings within a therapeutic context. A key step to testing this aspect of theory is to use experimental procedures where the source of the emotionally stimulating moment is standardized (Anderson et al. 2020), and the measurement of therapist emotions are near to their spoken interventions. However, there is little empirical evidence establishing this hypothesized link between therapist emotions during difficult therapeutic moments and therapist skill.

Only a few studies have begun to address how therapists' experiences might influence their responses on the FIS task. Stiles (2023) proposed the FIS task can contribute to the development of a theory of therapist responsiveness, which includes two basic notions of 1) those aspects from themselves therapists are *responding with* and 2) the characteristics of the simulated clients in the videos therapists are *responding to*. Thus, understanding therapists' emotions is theoretically a distinct part of the task. Antebi‐Lerman (2024) used a method of collecting therapists' responses to the FIS‐simulated clients and synchronizing these with therapists' ratings of their feelings during the intervention. Experimentally synching the difficult moment of the intervention with therapists' self‐reported feelings further removed measurement errors in retrospective recall of feelings during these difficult moments with clients. Using this method, Antebi‐Lerman identified significant therapists' some emotions (specifcially, emotional confidence and inadequacy) within moments of having responded to clients who had expressed in‐session emotional connection challenges.

## 1 Therapist FIS, Emotional Responsiveness, and Challenging Therapy Moments

As discussed, the FIS task exposes participants to simulations of therapy where they are to respond to standardized, realistic, and challenging client stimuli. In theory, the FIS task's isolation of difficult scenarios should create more opportunities for helpers to express hostility, make errors, and have more interpersonally problematic reactions than what occur and can be observed in typical therapy situations. In other words, the FIS task simulates especially urgent emerging contexts (Stiles 2021) in which a helper is put on the spot to “do the right thing” (Anderson et al. 2020) and show interpersonally appropriate responsiveness (Kramer and Stiles 2015) within the difficult therapeutic situation. A low FIS helper is, therefore, one that is thought to be less able to manage the stressors and interpersonal demands of the scenarios, resulting in “ballistic” (i.e., insensitive and non‐responsive) communication with the client (Stiles 2021). Critically, helpers who have difficulty expressing non‐reactive reflexivity and relative comfort to challenging moments with clients may see those clients experience poorer outcomes too.

Unfortunately, research directly evaluating whether helpers respond to more difficult simulations with more problematic experiential reactions and less skill (FIS) is quite limited. In one study examining the effects of training in interpersonal skills, Perlman et al. (2020) found that students experienced increases in positive and affiliative emotions while they were responding to the FIS clips, while the task had no significant influences on negative emotions. However, questions of whether more or less difficult stimuli truly produce differing experiential responses and whether the presence of more positive or negative emotional reactions predict performance on the task have not yet been empirically assessed.

Thus, understanding the emotional experiences of therapists during difficult moments should provide important information about how therapists are skillfully responsive to clients. Anderson et al. (2020) theorized that therapist interpersonal skill during the FIS task can be explained by how therapists respond to their own negative emotions while responding to objectively difficult client moments. Consistent with previous findings and clinical theory on how therapists resolve alliance ruptures (Muran and Eubanks 2020) and navigate difficult countertransference (Hayes et al. 2018), it seemed plausible that the level of difficulty found in an FIS‐simulated client would be associated with therapists' skills. That is, it would make sense that with more provocative and difficult situations, skillful therapists would have relatively fewer negative emotions. Furthermore, less difficult, or benign, therapy situations should have relatively less therapist negative emotion for therapists with higher FIS.

## 2 The Present Study

To explore possible differential responses on the basis of difficulty presented by clients, we prepared ‘benign’ FIS clips in which the same simulated client presents with low difficulty. These “benign” clips were prepared as comparisons to the standard FIS clips that were designed to have high interpersonal difficulty and are, subsequently, more emotionally provocative. Thus, this study examined whether more difficult therapeutic situations differ from less difficult situations in terms of the helper's self‐reported experiential responses immediately after viewing and responding to the simulated clients. We also aimed to test a model where therapists' self‐reported emotions predict the observable skill (FIS). We further aimed to evaluate whether and how different experiential responses would affect helpers' self‐perceived skills. Specifically, we hypothesized that (1) helpers would demonstrate higher skill levels (FIS) for benign clips than for difficult clips; (2) helpers would report different experiences when responding to difficult and benign video clips; (3) helpers' experiences when responding to clips would predict the observer‐rated skills (FIS) and the self‐reported skills (FIS); and (4) in‐task experiences would mediate the association between empathy and FIS.

## 3 Methods

### 3.1 Participants

Participants were recruited from a university research participant pool where all 96 participants responded to an advertisement that targeted involvement from those who were interested in pursuing a helping occupation; this study was described as one that would be appealing to those who might be interested in a future career in psychotherapy or as a mental health service provider. College student pools of interested helpers have been sampled previously to model and test clinically relevant uses of the FIS, such as how repeated modeled practice using the clips can improve skill (Anderson, et al. 2019). Participants were asked to select which helping occupation they were interested in pursuing (in the future), and more than one occupational interest could be selected. Approximately 31% of the sample indicated interest in becoming a counselor, 27% reported a desire to become a clinical psychologist, and 26% detailed social work as an occupational interest. Moreover, interest in pursuing a career as a mental health technician/worker was indicated by almost 22% of participants, and psychiatry was a potential occupational pursuit for approximately 19% of the sample. The participants were primarily White (84.4%) and female (60.4%) with a mean age of 19.19 years.

### 3.2 Measures

#### 3.2.1 Observational Judgments of Skill (FIS Ratings)

One of the primary dependent variables within this study was an objective rating of FIS, which was represented by an observational judgment of participant skill. Specifically, text‐based FIS judgment—or coding—was utilized for this study. Four judges coded each text‐based response (to video stimuli) collected under the Facilitative Interpersonal Skills Performance Task (Anderson et al. 2009). Each judge was trained for text‐based FIS coding over an approximately 3 month period, wherein coding assignments were completed and discussed among all four judges on a bi‐weekly basis. Each response was scored based on eight items reflecting the following facilitative conditions: warmth, acceptance, and understanding; empathetic accuracy; alliance‐bond capacity; verbal fluency; emotional expression; persuasiveness; hopefulness; and alliance rupture‐repair responsiveness. A 5‐point Likert scale was used, ranging from the presence of skill deficits (‘1’) to the optimal presence of a skill (‘5). The raters used a rating of 3 as a baseline and increased or decreased ratings based on the evidence of skills present or lacking throughout participants' responses to the video stimuli. The means of all coders' ratings across all eight items comprised the final FIS scores for each response. An inter‐rater ICC of 0.932 was achieved.

#### 3.2.2 Self‐Report FIS Judgement

Participants' perceptions of their own success at the FIS task were also recorded in the study. After completing all FIS performance tasks, participants were asked to complete a questionnaire regarding how well they believed they performed on the FIS task. Participants assessed their own response performance by completing eight items on a 5‐point Likert scale. They were asked to indicate how much eight different statements reflected their experiences with the responses they gave the clients in the video clips. For example, participants were asked to indicate the extent to which they disagreed or agreed with the statement that they believed their responses would have helped the clients. They also rated the extent to which they believed they provided warmth and understanding toward the clients. The self‐report FIS questionnaire was completed at the end of all of FIS performance tasks.

#### 3.2.3 Interpersonal Reactivity Index (IRI)

The Interpersonal Reactivity Index (IRI; Davis 1983) is a self‐report measure consisting of 28 items regarding participants' thoughts and feelings in a variety of situations. Each item was rated on a 5‐point Likert scale, ranging from 0 (‘does not describe me well’) to 4 (‘does describe me very well’). The IRI consists of four subscales, each composed of seven items: Perspective Taking, Fantasy, Empathetic Concern, and Personal Distress. The Perspective Taking and Fantasy subscales measure the cognitive aspects of empathy, whereas the Empathic Concern and Personal Distress subscales measure the emotional aspects of empathy. The sum of these subscales provides an overall estimate of trait empathy, with total scores ranging from 0 to 112. Davis (1983) reported that all four subscales were positively correlated with other measures of empathy, suggesting concurrent validity. Davis (1980) reported internal consistency (alpha) estimates ranging from 0.71 to 0.77, and test‐retest reliability from 0.62 to 0.71.

#### 3.2.4 Negative Affect and Positive Affect

The Positive and Negative Affect Schedule (PANAS; Watson and Clark 1988), comprised of two self‐report 10‐item mood scales, and is a self‐report measure of negative affect (NA) and positive affect (PA) during researcher selected time intervals. The 20 items are rated with a 5‐point Likert‐type scale with “1” representing “very slightly or not at all” and “5” representing “extremely”. For the present study, participants were instructed to rate the time period during which they provided their interventions to the FIS clips. The total score of PA and NA was calculated by summing the 10 positive items and the 10 negative items respectively. Both NA and PA subscales demonstrated acceptable reliability for the current sample (NA: *α* = 0.76; PA: *α* = 0.77).

#### 3.2.5 Self‐Report State Anxiety

The State Trait Anxiety Inventory (STAI; Spielberger 1983) is a self‐report measure of the state of subjective anxiety, and the current study used a six‐item version developed by Tluczek et al. (2009). The six‐item version of STAI demonstrated good psychometric properties and was found to be highly correlated to the full scale STAI (Tluczek et al. 2009). Participants were asked to rate each item on a four‐point Likert‐type scale (1 = ‘not at all’; 4 = ‘very much so’). The reliability for the current sample was good (*α* = 0.82).

### 3.3 Procedures

Prior to completing the experimental task, participants responded to items from the Interpersonal Reactivity Index (IRI) to provide trait‐level information. Next, the Facilitative Interpersonal Skills (FIS) Performance Task was completed. In the Facilitative Interpersonal Skills (FIS) Performance Task (Anderson et al. 2009), participants were asked to respond to a series of video clips depicting challenging (e.g., clients being too friendly, hostile, controlling, and submissive) and benign scenarios in therapy. Each simulated client was shown in full view and the shoulder of a therapist was visible in the corner of the screen. At the end of each client statement, the vignettes were paused so that participants could respond as if they were the therapist.

Each video clip was approximately 45 to 60 s long, and participants responded to a total of eight video clips from four different clients (two males and two females). One difficult and one benign clip for each client were included; “difficult” stimulus clips involved a reference to the therapist with some form of request (even if the request was merely implied). Difficult clips may have also included challenging interpersonal and emotional messages from the client, whereas “benign” stimuli were judged to be ordinary by coders. These clips also featured clients focusing on their issues without including particularly challenging messages on behalf of the client. Two different versions of video clip ordering were presented, and participants were randomly assigned to the different ordering presentations. One ordering group was exposed to four benign clips and then four difficult clips, while the other group was exposed to four difficult clips before four benign clips. At the end of each client statement, participants were told, “It's Your Turn to respond to this client. Please type in the text box what you would actually say to this client right now.” Individuals then typed out their responses to each stimulus clip.

After responding to each FIS clip, participants completed state experience measures representing positive affect, negative affect, and state anxiety. When the FIS task was completed, post‐experimental measures were completed. Participants indicated how well they believed they performed on the FIS task by completing the self‐report FIS judgement measure. Actual FIS scores for each clip were coded by four judges over an approximately 3‐month period.

### 3.4 Data Analysis

Paired *t*‐tests were performed to compare helpers' FIS scores and in‐task experiences (i.e., PA, NA, state anxiety) in difficult clips to those in benign clips. Then, helpers' FIS scores were regressed on their in‐task experiences to examine predictors of actual and self‐report FIS. Finally, a mediation analysis was performed to examine the roles of in‐task experience as mediators of association between empathy and FIS. The in‐task experiences (i.e., PA, NA, state anxiety) that were found to be significantly associated with FIS will be included as mediators.

## 4 Results

Table 1 presents correlations, means, and standard deviations for the measured variables. FIS scores were found to be positively associated with empathy and positive affect while negatively associated with state anxiety. No association was found between FIS scores and negative affect.

**Table 1:** Correlation matrix of measured variables.

Variables	M	SD	1	2	3	4	5	6
1 Actual FIS	2.739	0.513		0.431**	0.231*	0.337**	−0.161	−0.240*
2 Self‐report FIS	3.708	0.784			0.281**	0.405**	−0.270**	−0.528**
3 Empathy	67.115	10.716				0.297**	−0.258*	−0.261*
4 Positive affect	26.634	8.093					0.113	−0.447**
5 Negative affect	14.987	5.847						0.591**
6 State anxiety	10.771	2.899						

* *p* < 0.05

** *p* < 0.01.

### 4.1 Do Difficult Clips Differ From Benign Clips?

Table 2 shows the differences in helpers' FIS scores and experiences when responding to difficult and benign clips. While the FIS ratings for benign clips were highly correlated with FIS ratings for difficult clips (*r* = 0.913, *p* < 0.001), helpers showed significantly higher FIS response to benign clips than to difficult clips (*p* < 0.001). Furthermore, helpers experienced higher levels of negative affect (*p* = 0.007) and state anxiety (*p* = 0.008) when responding to difficult clips than to benign clips. No difference was found in helpers' positive affect between benign and difficult clips.

**Table 2:** Comparison between difficult versus Benign clips.

Variables	Difficult clips		Benign clips			
	Mean	SD	Mean	SD	*t*	*p*
Negative affect	15.28	5.85	14.69	6.03	−2.78	0.007**
Positive affect	26.58	8.38	26.69	8.01	0.42	0.676
State anxiety	10.98	3.02	10.56	2.98	2.73	0.008**
Actual FIS	2.66	0.53	2.82	0.51	6.78	< 0.001***

** *p* < 0.01

*** *p* < 0.001

### 4.2 Do Helpers' Experiences When Responding to Clips Predict FIS?

Helpers' positive affect was found to be a significant predictor of their actual FIS (*b* = 0.027, *p* = 0.002) and self‐report FIS (*b* = 0.024, *p* = 0.033). Helpers who reported higher levels of positive affect showed higher levels of actual FIS and self‐report FIS. In contrast, negative affect was not associated with actual FIS or self‐report FIS. Additionally, state anxiety was found to be negatively associated with helpers' self‐report FIS.

### 4.3 Do Helpers' Experiences Mediate the Association Between Empathy and FIS?

The direct associations between empathy and FIS in the absence of mediators were significant for both observer‐rated FIS (*b* = 0.011, *p* = 0.29) and self‐report FIS (*b* = 0.22, *p* < 0.001), suggesting that helpers with higher levels of empathy showed better helping skills when responding to therapeutic clips. Mediation models were performed to determine whether the associations between empathy (total score and subscales) and FIS were mediated by helpers' experiences when responding to therapeutic clips. Given that positive affect and state anxiety were associated with FIS scores while negative affect was not associated with neither observer‐rated FIS and self‐report FIS, we included positive affect and state anxiety as mediators.

**Figure 1:**
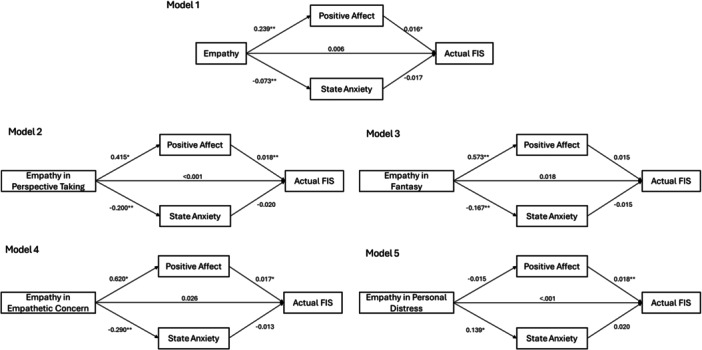
Mediation models of actual FIS*. Note:* Model 1: *R* ^2^ = 0.104 for positive affect; *R* ^2^ = 0.075 for state anxiety; *R* ^2^ = 0.122 for actual FIS; Model 2: *R* ^2^ = 0.054 for positive affect; *R* ^2^ = 0.095 for state anxiety; *R* ^2^ = 0.103 for actual FIS; Model 3: *R* ^2^ = 0.124 for positive affect; *R* ^2^ = 0.081 for state anxiety; *R* ^2^ = 0.137 for actual FIS; Model 3: *R* ^2^ = 0.058 for positive affect; *R* ^2^ = 0.098 for state anxiety; *R* ^2^ = 0.135 for actual FIS; Model 5: *R* ^2^ < 0.001 for positive affect; *R* ^2^ = 0.038 for state anxiety; *R* ^2^ = 0.098 for actual FIS. *p < 0.05, **p < 0.01, ***p < 0.001.

As shown in Figure 1, empathy was found to be indirectly associated with observer‐rated FIS via positive affect. Specifically, helpers with higher levels of empathy were more likely to experience positive affect (*b* = 0.239, *p* < 0.01) and less likely to experience anxiety (*b* = −0.073, *p* < 0.01) when responding to therapeutic clips, and, subsequently, positive affect was associated with higher observer‐rated FIS (*b* = 0.016, *p* < 0.01). Similar patterns were found for empathy in perspective taking and empathetic concerns. Helpers with higher levels of empathy in perspective taking were more likely to experience positive affect (*b* = 0.415, *p* < 0.05) and less likely to experience anxiety (*b* = −0.200, *p* < 0.01) when responding to therapeutic clips, and, subsequently, positive affect was associated with higher observer‐rated FIS (*b* = 0.018, *p* < 0.01). Helpers with higher levels of empathy in empathetic concerns were more likely to experience positive affect (*b* = 0.620, *p* < 0.05) and less likely to experience anxiety (*b* = −0.290, *p* < 0.01) when responding to therapeutic clips, and, subsequently, positive affect was associated with higher observer‐rated FIS (*b* = 0.017, *p* < 0.01).

**Figure 2:**
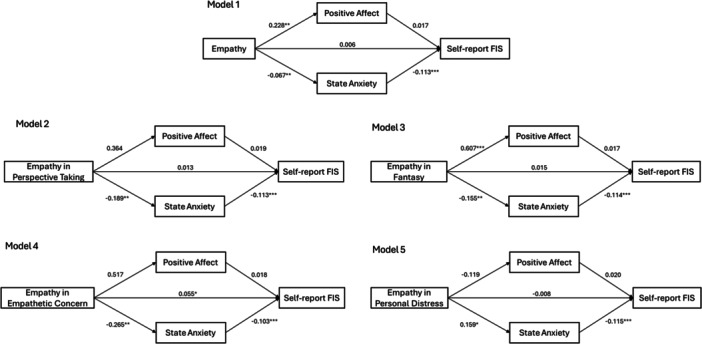
Mediation models of self‐report FIS*. Note:* Model 1: *R* ^2^ = 0.091 for positive affect; *R* ^2^ = 0.063 for state anxiety; *R* ^2^ = 0.286 for actual FIS; Model 2: *R* ^2^ = 0.041 for positive affect; *R* ^2^ = 0.087 for state anxiety; *R* ^2^ = 0.269 for actual FIS; Model 3: *R* ^2^ = 0.147 for positive affect; *R* ^2^ = 0.076 for state anxiety; *R* ^2^ = 0.284 for actual FIS; Model 4: *R* ^2^ = 0.039 for positive affect; *R* ^2^ = 0.081 for state anxiety; *R* ^2^ = 0.317 for actual FIS; Model 5: *R* ^2^ = 0.004 for positive affect; *R* ^2^ = 0.051 for state anxiety; *R* ^2^ = 0.256 for actual FIS; *p < 0.05, **p < 0.01, ***p < 0.001.

Conversely, the association between empathy and helpers' self‐reported FIS was mediated by state anxiety (Figure 2). Helpers with higher levels of empathy were more likely to experience positive affect (*b* = 0.228, *p* < 0.01) and less likely to experience state anxiety (*b* = −0.067, *p* < 0.01) when responding to therapeutic clips, and subsequently, decreased state anxiety was associated with higher levels of self‐reported FIS (*b* = −0.113, *p* < 0.001). The pattern remained consistent for empathy in perspective taking, empathy in fantasy, and empathy in empathetic concern, but not for empathy in personal distress. Specifically, helpers with higher levels of empathy in perspective taking, empathy in fantasy, and empathy in empathetic concern were less likely to experience state anxiety (*p*s < 0.05) when responding to therapeutic clips, and subsequently, decreased state anxiety was associated with higher levels of self‐reported FIS (*p*s < 0.01). In contrast, helpers with higher levels of empathy in personal distress were more likely to experience state anxiety (*p*s < 0.05) when responding to therapeutic clips, which led to lower levels of self‐reported FIS (*p* < 0.001). The direct associations between empathy and FIS (observer‐rated and self‐reported) were not significant in all models.

## 5 Discussion

As expected, there were significant differences in helper interpersonal skills between the difficult and benign situations even though the correlation between the two was strong. That is, interpersonal skills were separate, but related; they varied in response to the difficulty of the situation, but also remained highly consistent within helpers. Helpers' emotional experiences during these brief interventions predicted their level of interpersonal skills, but these predictions differed when skills were judged by the helper (‘helper skills’) versus when skill judgments were rated by independent observers using the FIS manual (“actual skills”).

### 5.1 State‐Level Experiences During Interventions

These findings provide a unique window into the helpers' immediate affective experiences when giving a brief intervention to a challenging client. From the helpers' perspective, beliefs about having higher interpersonal skill while responding were most predicted by lowered anxiety (or conversely, higher state anxiety predicted lower skill). This finding seems relevant to numerous findings that suggest that one major purpose of clinical supervision, especially during initial training, is to reduce anxiety and stress and increase general comfort among supervisees (Bernard and Goodyear 2019; Koçyiğit 2023) or even to normalize anxiety within the helper's role (Borders 2009). It makes sense that helpers had such a high link between deactivated anxiety during the response and their later beliefs about their interpersonal skills.

Interestingly, helpers' anxiety was not predictive of ratings of interpersonal skill rated by independent observers (i.e., ‘actual FIS’). Instead, it was helpers' state level *positive* affect that was highly predictive of these actual FIS ratings. The finding is even more notable since the FIS clips were selected from the most difficult therapeutic situations (found from real therapeutic scenarios) to be highly challenging to the helper (Anderson et al. 2020). Similarly, negativity within psychotherapy processes was identified as an important moment of alliance rupture that called for therapist repair interventions (Safran and Muran 2000; Eubanks et al. 2019). The inference for choosing such “difficult situations” in psychotherapy, then, was that therapist reactions during critical events would be especially useful for identifying the most skillful therapists.

However, the present findings give us pause in assuming that difficult clips and helper negative and anxious reactions are unique to identifying helpers' interpersonal skills. First, the observer‐rated FIS was only marginally predicted by the helpers' lowered negative affect, though there was some influence, and there was no relationship to the helpers' state anxiety. Instead, the present findings suggest that observer‐ratings of skill were especially notable for how **positive** feelings during the intervention predicted interpersonal skill. It is reasonable to suggest that high levels of interpersonal skill were accompanied by increased activation—but of a *positive* nature! Second, the overall level of helpers' experiences of positive and negative affect was significantly different between the difficult and benign clips, albeit there were pronounced differences in anxiety levels on the difficult clips.

The findings are unique in that there is little to no research that has examined the helpers' immediate experiences during interventions as well as how those experiences predict skill.

Therapist emotional awareness has been long recognized as an important therapist characteristic, defined as both skillful (Lane and Smith 2021) and trait‐like characteristics—and even suggested as an expression of therapist emotional intelligence (Kaplowitz et al. 2011). Still, identifying therapist emotional state‐level contributions has been mired in the methodological difficulties of accessing state‐level experiences within psychotherapy sessions, except via observational ratings. In a study that examined the effects of training helpers in interpersonal skills, Perlman et al. (2023) found that students experienced increases in positive and affiliative emotions while they were responding to the FIS. Further, Perlman et al. found no significant influences of FIS responses on negative emotions. The present study replicates and extends those findings. We similarly found that increased positive (and not negative) emotions were implicated *during* interpersonally skillful responses.

While Perlman et al. found that targeted training increased positive feelings in skillful responding, the present study differed, because we showed that that positive affect *during* the helpers' response predicted the skill of responses—even without training. Taken together, both studies provide strong and consistent support for the benefits of helpers' positive affect during difficult therapeutic situations. Additionally, these studies lack evidence of what we once thought was the important role of the suppression, control, and reduction of negative experiences during difficult moments in psychotherapy.

Nonetheless, creative statistical strategies have demonstrated that it is a good idea to separate state from trait characteristics within psychotherapy. These contributions of state‐level characteristics have proven to be significant predictors of outcome, unique from trait‐ and person‐level predictors. A series of recent studies by Zilcha‐Mano and colleagues (e.g., Zilcha‐Mano and Fisher 2022) have successfully separated the trait‐like and state‐like characteristics of processes like the therapeutic alliance, indicating that state‐like characteristics of these processes were uniquely predictive of psychotherapeutic success. Understanding the contributions of momentary emotional states to skillful performance requires invasive procedures that would disrupt the ecology of the therapeutic environment. The value of simulative procedures used in the present study is that it presents a realistic, but still artificial, window into how therapists' momentary emotions impact levels of skillful performance. Examining the actual states within psychotherapy moments may be especially revealing, as we found strong associations between these state characteristics of the helpers and their skill level.

The helpers' pre‐existing trait characteristics did not have a direct influence on skillful expressions in response to these simulated clients. It is interesting that prior attempts to identify trait‐level predictors of skills only have been mildly successful. There have been only mild correlations between trait‐level predictors and both helping skills (Hill et al. 2016) and facilitative interpersonal skills (Anderson et al. 2020). Gumz et al. (2023) also have yielded mild associations between helper traits and psychotherapy processes and outcome. As summarized by Lingiardi et al. (2018), therapist subjective characteristic variables have an inconsistent record in predicting outcome, and therapist skill has more promise as a direct predictor. Fitting within these findings about therapist traits as predictors, the present study showed that there was an *indirect effect* of these trait‐level predictors, but via the state‐level experiences of helpers. Specifically, helper trait empathy predicted helpers' experiences of positive affect during the intervention, which in turn influenced skillful expression of the response. One possibility is that helper traits may have some effect on the ability of the helper to become *experientially responsive within critical helping situations*. Some traits may better prepare helpers to experience emotions that prepare them for more helpful interventions. It may be that training therapists to **actively** engage in positive feelings during challenging moments may be more beneficial than suppressing negative reactions. While this study does not address any causal relations, it's clear that empathy, positive affect, and actual observer‐rated FIS share a meaningful associative pathway.

This explanation fits within the small, but growing set of recent findings that have emerged in identifying nuanced and complex relationships when examining the role of therapist traits on skill—particularly interpersonal skills—and psychotherapy outcomes. Heinonen and Nissen‐Lie (2020) suggested that therapist traits which are derived from their personal lives and attachment histories, have been useful predictors and are meaningfully linked to the professional development of similar interpersonal characteristic, which are then expressed as effective interpersonal skills. Recent findings by Delgadillo et al. (2020) have found that therapist Big 5 personality traits on agreeableness and openness predicted client outcomes, but that these traits were unique to either well‐being counselors or cognitive interventionists. In some cases, the link between therapist traits and skill is stronger, but so too is the definitional murkiness of the trait, such as Delgadillo et al. (2018) finding that therapist burnout predicted therapy outcomes. Trait empathy in the present study is similarly an interpersonal characteristic that is conceptually linked to professionally relevant skills that can be developed, while still defined as a trait and hence conceptually grounded in the helpers' background. The present study fits within this literature, showing that a trait (empathy) that is conceptually close to the skill (FIS) can be predictive, albeit as an indirect effect and via the helpers' experiences.

### 5.2 Limitations and Future Directions

There are several limitations and future directions in the present study. First, helpers' self‐report of in‐task experiences may not match their genuine subjective experiences or beliefs about their interpersonal skills. Second, this study was based on simulations. While experimentally useful for understanding theory, there are several ways the study may not match clinical realities. For instance, compared to self‐identified “helpers,” real therapists in practice may react differently and likely more skillfully. Third, our study was based on cross‐sectional data, which limits the ability to draw causal inferences about the relationships between empathy traits, emotional reactions, and FIS. It would be worthwhile for future researchers to replicate our findings using a longitudinal design.

Fourth, we only included limited number of emotional reactions during FIS task. Future studies should consider examining a broader range of emotional experiences, such as frustration and helplessness, to better detect the complexity of helpers' emotional reactions during challenging therapeutic interactions and how their emotions impact their skills. Finally, this study used text‐based responses, which may not match actual verbal responses. In terms of future study, it's worth looking for therapist traits that might facilitate the development of therapist skills. Based on these findings, it's natural to think that helpers' dispositional positivity might be a good predictor. Finally, it would be interesting to see if training therapists to maintain positive experiences during difficult moments—or *any* interpersonal situations—may be a way to increase clients' interpersonal skills in training and supervision.

## Ethics Statement

These data were collected with the approval of our Institutional Review Board. All research procedures followed this approved protocol from Ohio University IRB. Anonymized data for this study are available to qualified researchers upon request. All inquiries should be directed to the first author at andersot@ohio.edu or Department of Psychology, 215 Porter Hall, Ohio University, Athens, Ohio 45701.

## Data Availability

The data that support the findings of this study are available on request from the corresponding author. The data are not publicly available due to privacy or ethical restrictions.
